# "Heart Failure: Meeting the Challenges of Surveillance and Knowledge Translation in Resource-poor Settings"

**DOI:** 10.2174/1573403X11309020002

**Published:** 2013-05

**Authors:** Shahulhameed Safraj, Vamadevan S Ajay, Dorairaj Prabhakaran

**Affiliations:** 1 CoE, CARRS, Public Health Foundation of India, New Delhi; 2 Centre for Chronic Disease Control, New Delhi

**Keywords:** Heart failure, surveillance, knowledge, translation.

## Abstract

Heart failure (HF) is a major cause of morbidity and mortality in high income countries. Shortage of population based HF studies from Low and Middle Income countries (LMIC) make global prevalence estimates difficult. In this editorial we discuss the possibility of generating HF data in LMICs by initiating HF surveillance systems integrated into the existing health surveillance system..

## INTRODUCTION

Heart failure (HF) is one of the major causes of morbidity and mortality in high income countries with around 2 % of adults suffering from heart failure [1]. Estimation of the burden of Heart failure is more complicated in developing countries due to reasons like absence of proper surveillance systems and lack of a standard definition. Shortage of population based HF studies from developing countries makes global prevalence estimates difficult. A report from India estimated that HF due to CHD, hypertension, obesity, diabetes and RHD in 2000 ranged from 1.3 Million to 4.6 Million [2]. With increase in the number of aged people and in the prevalence of risk factors in developing countries the magnitude of the problem is bound to increase significantly. Accessibility and affordability of treatments are also major issues in low resource settings. Given the age of patients with HF in developing countries is much lower than that in the west and with the population at risk being higher, the economic and social impact of HF in developing countries is enormous.

In this theme issue, experts from different regions of the world discuss heart failure in their geographical context with an updated review of current literature regarding epidemiology, pathophysiology, prevention, management and future challenges.

Harikrishnan in his review about Heart failure in South Asia reports that the aetiology of HF in the region is different from the west with Rheumatic Heart disease and congenital heart disease contributing significantly. There is also difference of features of HF in south Asians versus whites such as Lower age of presentation and age adjusted mortality, ischemic etiology of HF, higher chances of history of MI prior to the first HF admission and increased hospital readmissions. Diabetes in HF patients is more common where as Atrial fibrillation is less common. Al Shamiri in his review about Heart failure in Middle East notes on the recent progress in the area with the development of regional and multicentre registries, however he also reports on the lack of comprehensive data from the region. He suggests that the difference in the prevalence of diastolic HF in Middle Eastern studies may be due to Selection bias or the inaccurate use of ECG diagnostic criteria. He also reports that in the most of Middle Eastern countries more than half of HF cases are secondary to IHD. Bochi , reports that in South America, the main etiologies of heart failure are ischemic, idiopathic dilated cardiomyopathy, valvular, hypertensive and chagasic etiologies with Chagas heart disease responsible for upto 41% of the HF cases in endemic areas. However, he observed a reduction of HF mortality due to Chagas heart disease from 1985 to 2006, and of mortality due to HF from 1999 to 2005 in selected states in Brazil.

From the available data it is clear that there is great variation in the prevalence and incidence of HF around the world and causes’ significant burden on the health system of nations. A recurring theme reported in these papers is the lack of adequate data from developing countries. Most of the knowledge about HF is derived from North American and European studies. In contrast, no population-based study has been reported from the developing world, and the scarce available information comes from data gathered in clinical trials or hospital-based studies. Therefore, it appears that the biggest challenge facing developing countries with limited resources is the generation of quality data about HF. The easiest way to generate HF data is by initiating a surveillance system for HF integrated into the existing health surveillance information system. 

An ideal surveillance system captures and tracks all important manifestations of a disease, providing key information on disease activity including persons affected, timing, magnitude, severity and location in order to guide implementation of medical and public health measures to control or contain the disease [3]. Similarly, a HF surveillance system should include burden (incidence and prevalence), awareness, risk factors, health consequences, processes and quality of care, and health care system capacity. The burden of disease should be described according to stages of HF and within different demographic (e.g., age, sex, race, and ethnicity) and clinical (e.g., diabetes and hypertension) groups, across different geographic areas (e.g., states) and across time.

The main challenge would be to develop a surveillance system that is simple, flexible, sensitive, representative, timely, stable and acceptable. The system has to be structurally and operationally simple so that it can be carried out with minimal additions to the existing surveillance systems and within the limited resources. The system should be flexible enough to look at new questions posed by research and accommodate changes in technology or reporting definitions. It should be able to generate complete and valid data and be sensitive enough to capture all events and monitoring trends and have a high positive predictive value to capture all true cases. The system should also be representative and acceptable enough to generate enthusiasm and willingness to participate and be stable enough to be operated reliably and provide required information in a timely fashion. Conceptual and methodological challenges such as sampling, establishing a population denominator, geographical data for state and local planning, standardisation of measures, variable definition of measures and variability in data quality have been described as challenges in establishing surveillance systems [4]. In settings with the limited resources the situation is further complicated by the lack of data from the national, state and local levels, shortage of trained staff, lack of investigative facilities and absence of strict diagnostic criteria.

The first step in the development of a surveillance system should be to develop a vision of a ‘future proof’ surveillance system. A ‘future proof’ surveillance system is a surveillance system that is developed in the present with the aim on how it would work in the future. The vision should be developed based on the sources of the surveillance data, methods of surveillance, reporting standards, level of health system integration and capacity. Once this has been completed then the steps needed to actualize this vision needs to be theorized and the financial resources procured. States could start by establishing the data needs, identifying and training the staff, decide on the instruments and data management process. Many types of data sources could provide relevant data for a HF surveillance system. These include population-based survey data, public and private health care system data, screening activity data, professional societies, private industry, cohort studies and registries. Developing a standard dataset, along with a standardized method for collecting data would be the first step in plan collating data from such multiple sources. Table **1** lists the major challenges and potential solutions with examples. 

One of the most consistent findings from clinical and health services research is the failure to translate research into policy. A closer relationship between research, policy and practice is needed to ensure that the knowledge generated is transferred into action. Though, the transfer of knowledge from field is complex and dependant on local conditions this transfer can support the introduction of interventions with proven efficacy. In many cases, the success of knowledge transfer depends on researchers and health care practioners changing their outlook and deciding on the process and mechanism through which the knowledge generated can be best transferred into practice. Studies suggest that the basic unit of knowledge translation be up to date systematic reviews or other synthesis of research findings. There are many different knowledge translation models, derived from various settings, however most studies suggest that Knowledge translation is more likely to be successful if the strategy is informed by an assessment of the likely barriers and facilitators. To ensure that the knowledge generated is translated into practice it is important that there is prior understanding on what knowledge should be translated and how it should be transferred. There should also be clarity on who should do the transfer to whom and with what effect. More research is needed to identify the specific skill building/ training requirement to facilitate translation of knowledge in the health system of developing countries that vary widely in their features.

The rising burden of HF in developing countries calls for a systematic approach. Efforts to put in place intervention programmes should be complemented with a robust surveillance mechanism so as to monitor evaluate and guide policies and programmes. The Global STEP wise approach for NCD risk factor surveillance formulated by the World Health Organization (WHO) is a good template. Aimed at collecting data on risk factors comparable across variable sites in the world in a stepwise manner according to the complexities involved. The STEP wise format emphasizes on ‘core’, ‘expanded’ and ‘optional’ variables which provide a common platform for comparability and flexibility to include variables for local requirements [12]. In India, the feasibility of establishing surveillance for CVD risk factors at community levels has been demonstrated in a pilot programme and the programme has now been scaled up to the national level, and is now included in the National Programme for Prevention and Control of Diabetes, Cardiovascular Diseases and Stroke [13]. The programme aims at providing health promotion, screening for NCDs, setting up specialty clinics, re-orientating the health system towards NCDs, building capacities, and strengthening linkages between various stakeholders. The activities would be aimed at community level, workplace and school settings. The NCD risk factor surveillance being conducted under Integrated Disease surveillance project (IDSP) has now been incorporated as part of the programme. The programme would collect data, determine priorities, assist planning, evaluate interventions, guide health policies, monitor programme goals, foster research, assess and document in-time needs and serve in developing long-term strategies.

Prevention of HF is of vital importance in low resource settings as accessibility and affordability to treatments are a major issue in low resource settings. Control of risk factors, establishment of dedicated HF clinics and initialising nurse and primary health care worker based management programs are some of the steps outlined in the papers in this issues. To properly manage a long term condition like HF, physicians and health care staff should understand the needs and culture of the community and should have access to locally relevant data relating to the nature, progression and management of HF. A properly designed surveillance system with findings regularly translated into policy is the best weapon in the fight against Heart failure.

## Figures and Tables

**Table 1.** Challenges and Potential Solutions for Developing and HF Surveillance System in Developing Nations

Component	Challenges	Solutions	Examples
Diagnosis criteria	Lack of simple diagnostic tools for use at primary care level	Research to develop tools and their validation	Gothenburg Criteria for diagnosis of heart failure [5]
Data sources	Lack of data sources that aid in collation of representative data at the national level	Periodic population based surveys that uses standardized methods Hospital based registry involving both private and public sector Data from cohorts	Demographic and Health Surveys [6] Kerala ACS registry [7] CARRS cohort in India and Pakistan [8]
Human resources	Lack of trained staff	Recruitment, training and capacity building for physicians and non-physician health workers	mPower Heart project in Himachal Pradesh, India
Laboratory facilities	Lack of laboratory facilities to carry out diagnostic investigations	Developing portable easy to use point-of-care devices and their use	Swasthya slate [9]
Data management	Lack of trained data management tools and expertise including data transfer, storage, analysis and feedbacks	Building capacity to the existing surveillance infrastructure	INCLEN [10]
Translation	Linking surveillance information to health policies and programs	Involvement of Stakeholders and advocacy	Mauritius National Non Communicable disease programme [11]
